# The competitive athlete with type 1 diabetes

**DOI:** 10.1007/s00125-020-05183-8

**Published:** 2020-06-12

**Authors:** Michael C. Riddell, Sam N. Scott, Paul A. Fournier, Sheri R. Colberg, Ian W. Gallen, Othmar Moser, Christoph Stettler, Jane E. Yardley, Dessi P. Zaharieva, Peter Adolfsson, Richard M. Bracken

**Affiliations:** 1 School of Kinesiology and Health Science, Faculty of Health, Muscle Health Research Centre and Physical Activity & Chronic Disease Unit, York University, 4700 Keele Street, Toronto, ON M3J 1P3 Canada; 2 LMC Diabetes & Endocrinology, Toronto, ON Canada; 3 Department of Diabetes, Endocrinology, Nutritional Medicine and Metabolism, Bern University Hospital, University of Bern, Bern, Switzerland; 4 Team Novo Nordisk Professional Cycling Team, Atlanta, GA USA; 5 School of Human Sciences, Division Sport Science, Exercise and Health, University of Western Australia, Crawley, WA Australia; 6 Human Movement Sciences Department, Old Dominion University, Norfolk, VA USA; 7 Royal Berkshire NHS Foundation Trust Centre for Diabetes and Endocrinology, Royal Berkshire Hospital, Reading, UK; 8 Cardiovascular Diabetology Research Group, Division of Endocrinology and Diabetology, Department of Internal Medicine, Medical University of Graz, Graz, Austria; 9 Augustana Faculty, University of Alberta, Edmonton, AB Canada; 10 Alberta Diabetes Institute, Edmonton, AB Canada; 11 Women’s and Children’s Health Research Institute, Edmonton, AB Canada; 12 Department of Pediatrics, Stanford University School of Medicine, Stanford, CA USA; 13 Department of Pediatrics, The Hospital of Halland, Kungsbacka, Sweden; 14 Institute of Clinical Sciences, Sahlgrenska Academy, University of Gothenburg, Gothenburg, Sweden; 15 Applied Sport, Technology, Exercise and Medicine Research Centre (A-STEM), Swansea University, A111 Engineering East, Fabian Way, Crymlyn Burrows, Swansea, SA1 8EN UK

**Keywords:** Athlete, Carbohydrate, Competition, Continuous glucose monitoring, Exercise, Glucose, Insulin, Nutrition, Review, Sport, Type 1 diabetes

## Abstract

Regular exercise is important for health, fitness and longevity in people living with type 1 diabetes, and many individuals seek to train and compete while living with the condition. Muscle, liver and glycogen metabolism can be normal in athletes with diabetes with good overall glucose management, and exercise performance can be facilitated by modifications to insulin dose and nutrition. However, maintaining normal glucose levels during training, travel and competition can be a major challenge for athletes living with type 1 diabetes. Some athletes have low-to-moderate levels of carbohydrate intake during training and rest days but tend to benefit, from both a glucose and performance perspective, from high rates of carbohydrate feeding during long-distance events. This review highlights the unique metabolic responses to various types of exercise in athletes living with type 1 diabetes.

**Electronic supplementary material:**

The online version of this article (10.1007/s00125-020-05183-8) contains a slideset of the figures for download, which is available to authorised users.

**Figure:**
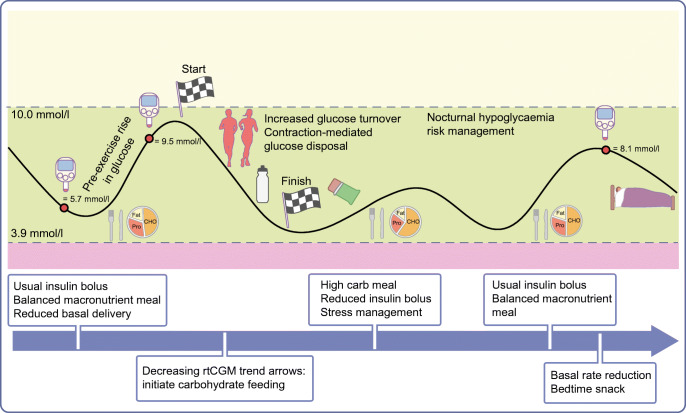
Graphical abstract

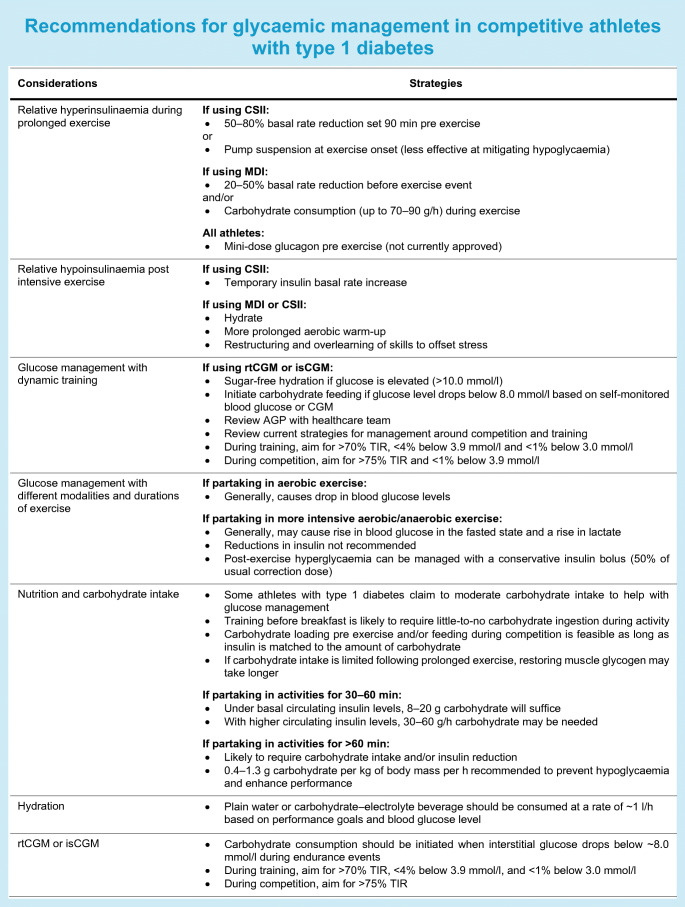



## Introduction

As we approach the 100-year mark of the discovery of insulin, people with type 1 diabetes may achieve a near normal life expectancy with an overall high quality of life, but this requires tight maintenance of on-target blood glucose levels and good cardiovascular health [1]. Both of these aspects of diabetes management are still very challenging for individuals with type 1 diabetes, even with access to specialised diabetes care [2]. Being regularly active with the disease improves cardiometabolic health [3] and is associated with increased longevity [4].

**Fig. 1:**
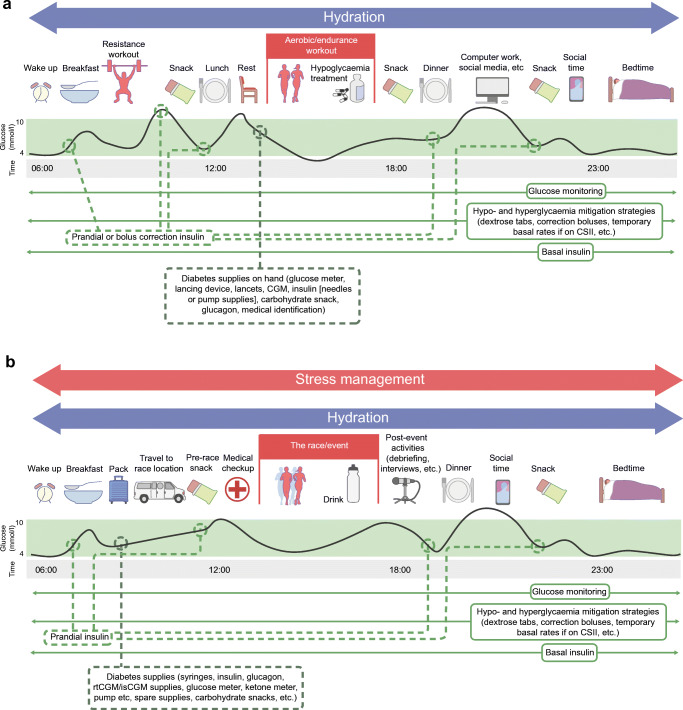
Example training day (**a**) and competition day (**b**) for a competitive athlete with type 1 diabetes. A number of variables need to be considered and controlled by an athlete with type 1 diabetes, including glucose monitoring, basal and bolus insulin-dose modifications, snacks and meals, hypo- and hyperglycaemia mitigation, hydration and stress management. Although some flexibility may be allowed on training days with regard to the timing of training and meals, this flexibility is lost on competition days due to strict competition schedules. Note that this is an example and will differ depending on numerous factors such as the event that the athlete competes in. This figure is available as part of a downloadable slideset

Leading up to the next Summer Olympic Games, numerous athletes with type 1 diabetes will train and compete at the elite level, with some aspiring to pursue their podium dreams. The day-to-day management of the condition remains onerous, however, given the monotonous tasks of monitoring glucose, carbohydrate/macronutrient counting, insulin dosing, and managing stress/sick days, particularly while training and preparing for competition (Fig. 1). Ongoing research is increasingly focusing on the unique physiology of such high-level athletes with type 1 diabetes, while also investigating how new insulin analogues and other therapeutic agents/technologies might improve their glycaemic management. This review highlights the challenges of high-level training and competition in athletes with type 1 diabetes and identifies some of the knowledge gaps that limit our capacity to provide evidence-based strategies to optimise their performance.

## Energy metabolism

Physical activity at all levels requires the mobilisation of various fuel sources. To help better understand the unique responses to exercise in type 1 diabetes, we briefly describe the main energy systems used during various forms of exercise in the following sections. Possible alterations in energy metabolism caused by the disease are highlighted.

### ATP and phosphocreatine

**Fig. 2:**
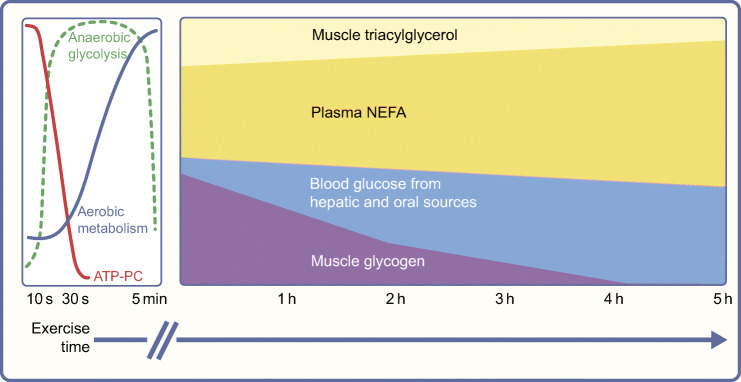
Energy substrates for exercise. The source of energy substrates during exercise varies depending on exercise duration. During skeletal muscle contraction, in the first few seconds of exercise, energy is provided from ATP, which is immediately resynthesised from phosphocreatine (PC). For exercise of longer duration, ATP resynthesis occurs by catabolising other fuel sources (lipids and carbohydrates). Figure based on previously published data [102, 103]. This figure is available as part of a downloadable slideset

During skeletal muscle contraction, energy is provided from ATP, which is immediately resynthesised from phosphocreatine. The limited phosphocreatine stores require that ATP resynthesis occurs by catabolising other fuel sources (lipid and carbohydrates) for exercise events lasting more than a few seconds (Fig. 2). With insulin therapy, and in the absence of nephropathy, ATP and phosphocreatine levels at rest and post exercise appear normal in individuals with type 1 diabetes [5]. However, a slower phosphocreatine recovery time and impaired mitochondrial function/capacity may exist in some individuals with the disease in whom blood glucose levels are not tightly managed with insulin therapy [5, 6]. Insulin deprivation and/or sustained hyperglycaemia can impair mitochondrial function, promote mitophagy, lower ATP provision and increase reactive oxygen species production in muscle, heart, kidney and brain [7].

### Carbohydrates

During high-intensity exercise, carbohydrate is a primary fuel source. Glucose stores within liver and skeletal muscle, in the form of glycogen, depend on the size and training status of the individual and are the body’s primary carbohydrate stores. In the average adult male weighing 70 kg, up to 160 g of glucose can be stored in the liver, while up to 700 g of glucose can be stored in the muscle [8]. A ‘normal’ blood glucose concentration of ~5–7 mmol/l amounts to only ~4–6 g of total blood glucose, depending on the person’s size. In individuals without diabetes, intense exercise causes a transient rise in glucose by ~2 mmol/l [9], while prolonged moderate-intensity exercise induces a small and transient drop in glucose by ~2 mmol/l [10], albeit responses are highly variable. Glucose production, predominantly by the liver via glycogenolysis and gluconeogenesis, as well as oral carbohydrates, help support normal blood glucose levels [11] (Fig. 2). Individuals with type 1 diabetes can have normal levels of muscle and liver glycogen content if they are adequately fed, take insulin and have good glycaemic control (HbA_1c_ <58 mmol/mol [7.5%]) [12, 13]. Hepatic glycogen levels are lowered by poor glycaemic control in individuals with type 1 diabetes [14], with only a partial restoration with short-term improvements in glycaemic control [15].

The flux of glucose from liver to muscle during exercise is impacted by insulin treatment, which can result in either hypo- or hyperglycaemia [16]. High insulin levels limit hepatic glucose mobilisation and increase muscle glucose disposal, thereby causing hypoglycaemia. Inadequate insulin levels cause hyperglycaemia, as glucose production exceeds utilisation [16].

### Lipids

Adipose tissue and skeletal muscle lipid stores are plentiful, even in lean individuals. Lipids are used heavily during prolonged exercise, particularly as the activity duration increases (Fig. 2). Peak absolute lipid oxidation rates occur at ~55–60% of maximal aerobic rate in trained individuals [17]. Intensive insulin therapy in type 1 diabetes often increases body fat stores and body weight [18], although this effect can be attenuated with dietary restriction [19] and/or endurance training [20]. Lipolytic potential may be elevated in type 1 diabetes, perhaps because of increased β-adrenoceptors on fat cells [21]. However, a high insulin level during exercise suppresses lipolysis/fat oxidation, as compared with basal insulin concentrations [22] (see below).

### Protein

Although protein is a major component of lean tissue, it does not normally contribute significantly to energy metabolism. However, some protein-derived amino acids, such as leucine or alanine, can contribute minimally to skeletal muscle energy needs, especially when carbohydrate availability is restricted (i.e. by low-carbohydrate diets, periods of insulin deficiency) [23]. The gluconeogenic conversion of protein-derived and free amino acids into glucose during exercise is upregulated in type 1 diabetes if insulin is withheld [24]. Insulin deficiency for as little as 8 h in type 1 diabetes, perhaps in combination with other factors (hyperglycaemia, elevated cortisol, inflammation, etc.), rapidly promotes protein catabolism, likely via activation of muscle-specific transcription factors [25].

## Insulin regulation and dysregulation during exercise

Insulin mediates glucose disposal into skeletal muscle and adipose tissue via increased glucose transporter type 4 translocation. In liver, insulin signalling supresses glucose production and activates glycogen synthesis via activation of various enzymes, including glucokinase and glycogen synthase [26]. During endurance exercise in individuals without diabetes, insulin secretion decreases via increased sympathoadrenal drive, with the magnitude of decline closely linked to activity intensity and duration [27]. This drop in insulin secretion facilitates lipid and glucose mobilisation from stores outside of the muscle, while minimising the risk for hypoglycaemia as contraction-mediated glucose disposal increases [16]. With brief intensive exercise bouts, insulin secretion increases during early recovery to offset rising glucose concentrations [9].

**Fig. 3:**
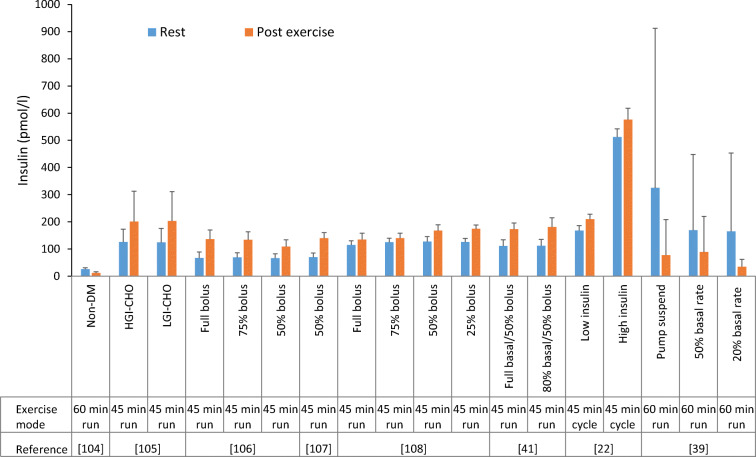
Circulating insulin levels in physically active individuals with type 1 diabetes. The values shown represent the insulin concentration as measured before (rest) and soon after the end of exercise in a variety of previously published studies, which included various cohorts/conditions: non-diabetic control participants (Non-DM); participants with type 1 diabetes who underwent high- (HGI-CHO) or low- (LGI-CHO) carbohydrate feeding interventions; participants with type 1 diabetes who underwent bolus insulin-dose reductions (full bolus; 75% bolus; 50% bolus; 25% bolus); participants with type 1 diabetes who underwent basal insulin-dose reductions, along with bolus dose reductions for MDI (full basal/50% bolus; 80% basal/50% bolus); participants with type 1 diabetes who had low (15 mU m^−2^ min^−1^) or high (50 mU m^−2^ min^−1^) intravenous insulin infusions; and type 1 diabetic participants who underwent basal insulin rate reductions for CSII (pump suspend; 50% basal rate; 20% basal rate). The mode of exercise and duration of activity is shown on the *x*-axis. Data are from select studies [22, 39, 41, 104–108] and were analysed by R. M. Bracken. This figure is available as part of a downloadable slideset

In individuals with type 1 diabetes, circulating insulin levels depend on the amount and location of insulin administration. Because insulin levels cannot immediately be lowered at exercise onset, individuals with type 1 diabetes are often hyperinsulinaemic during their activity (Fig. 3). The relative hyperinsulinaemia during prolonged moderate-intensity exercise supresses lipolysis/fat oxidation [22] while increasing whole-body glucose utilisation and hypoglycaemia risk [16]. Exercise increases absorption rates of some [28], but not all [29] forms of insulin, which can exacerbate the risk for hypoglycaemia. With intensive exercise, hyperglycaemia post exercise is aggravated by the inability to automatically increase insulin delivery into the portal circulation [9]. Omitting insulin altogether, well in advance of exercise, promotes excessive hyperglycaemia and ketone production [30].

## Selecting an insulin delivery method

The primary goal of exercise management in athletes with type 1 diabetes should be to limit dysglycaemia, with a secondary goal of attempting to replace insulin to healthy physiological insulin levels. Complete restoration of insulin to physiological levels is impossible since insulin is administered subcutaneously rather than released into the portal circulation. While some athletes with type 1 diabetes perform well using multiple daily injections (MDI) of insulin [31], others prefer the flexibility afforded by continuous subcutaneous insulin infusion (CSII) [32]. The latter allows for temporary basal rate reductions in anticipation of and/or recovery from prolonged aerobic exercise, temporary basal rate increases for very intensive aerobic/anaerobic work, and for basal rate reductions overnight, if nocturnal hypoglycaemia is an issue. Hybrid closed-loop technology may support glycaemic management in athletes better than traditional pump therapy as insulin delivery is informed by current glucose levels, glucose predictions, previous insulin delivery and other features of proprietary algorithms that improve overall ‘time in range’ (TIR; the percentage of time that an individual’s blood glucose is within the target level) [33]. Currently approved hybrid closed-loop devices are suitable for prolonged aerobic exercise if a temporary (higher) glucose target is set well before the start of exercise (i.e. 45–90 min before the exercise start time).

In spite of these benefits, many individuals report that CSII interferes with their sporting activities or that they would rather not be attached to a medical device [34]. Maintaining insulin infusion sets and glucose monitoring devices during exercise and sport is challenging when there is increased perspiration and the potential for sport contact and/or friction. For athletes who prefer pump removal during exercise, a hybrid approach that combines basal insulin delivery split between an ultra-long-acting insulin and 50% reduced basal insulin delivery by CSII [35] can be used. The addition of continuous glucose monitoring (CGM) is beneficial, as athletes (particularly those with hypoglycaemia unawareness [36]) can gather glucose data, respond to glucose trend arrows and alerts/alarms, and optimise therapy [37]. Real-time CGM (rtCGM) offers the advantage of alerts and alarms when glucose drifts away from target; however, exercise itself has an impact on sensor accuracy [38].

## Strategies to address relative hyperinsulinaemia during prolonged exercise

Relative hyperinsulinaemia during prolonged aerobic exercise can be offset by basal and/or prandial insulin-dose reductions and/or by increased carbohydrate feeding. For those using CSII, basal insulin delivery can be reduced by 50–80% 90 min before exercise [39]. Suspending insulin delivery at exercise onset is safe, albeit less effective in mitigating the drop in blood glucose level [40]. Basal insulin delivery can be resumed immediately post exercise, allowing circulating insulin levels to rise before the recovery meal.

For individuals using MDI, the basal insulin dose can be reduced by 20–50% before exercise to mitigate hypoglycaemia risk [41]. Even insulin degludec can be reduced by ~25%, but this reduction should be initiated 3 days before the exercise event [42]. For other long-acting basal insulins (e.g. insulin glargine, insulin detemir), the total basal insulin dose can be divided into a morning and evening dose to allow for more flexible adjustments. As an alternative (or complement) to basal insulin-dose reduction, simple carbohydrate consumption (up to 70–90 g/h) during prolonged aerobic activities can help prevent hypoglycaemia and support performance [43].

In addition to the inability to lower insulin secretion into the portal circulation at exercise onset, glucagon fails to rise normally during prolonged exercise in type 1 diabetes, predisposing athletes to developing hypoglycaemia during some activities [44]. Administering a mini dose of glucagon [45] or glucagon in a dual-hormone closed-loop pump [46] helps to eliminate hypoglycaemia; however, this has never been tested in a setting of competition.

## Strategies to address relative hypoinsulinaemia post exercise

Managing competition-related hyperglycaemia, particularly at the start of an event, can be challenging [47]. Psychological strategies, such as cognitive restructuring and overlearning of skills, may help offset the stress effects [48]. Some athletes will tolerate, or even plan for, a slightly elevated blood glucose level when starting an event; others may choose to use a temporary basal rate increase (if using CSII), a partial bolus insulin correction or a prolonged aerobic warm-up to correct hyperglycaemia. Giving a standard (i.e. full) insulin bolus correction before a prolonged aerobic exercise event is not recommended unless ketones are elevated, since doing so increases hypoglycaemia risk [11].

Many athletes have difficulty managing immediate post-event hyperglycaemia [42]. When fasted, a bolus insulin correction can be given after intense aerobic exercise [49] or after resistance exercise [50]. However, with most prolonged exercise events, late-onset hypoglycaemia remains common for athletes [51] and, thus, basal insulin-dose reduction and/or bedtime snack strategies are recommended [16].

## Planning for glucose management with dynamic training protocols

Athletes partake in varied training regimens, often differing daily or seasonally with regard to training mode, intensity and duration. Professional athletes frequently use ‘polarised’ training strategies, starting early in the season with low-intensity, high-volume work, followed by high-intensity, lower volume work later in the season. Before competition, training volume generally tapers. Such training varieties may make glucose management challenging for athletes with type 1 diabetes. However, by individualising standard recommendations, athletes can personalise strategies through trial and error to temper glycaemic excursions [47].

Even when athletes with type 1 diabetes have well-honed strategies, it is often useful to work with endocrinologists and other healthcare providers [47]. The clinical team should first review the ambulatory glucose profile (AGP) if rt-CGM or intermittently scanned CGM (isCGM) is used, along with the athlete’s current strategies for glucose management around training and competition. Clinicians should review glucose monitoring downloads to ensure adequate basal insulin dosing and correct bolus insulin usage to cover meals and hyperglycaemic excursions [52]. The clinical team should offer reasonable initial strategies for athletes who are newly diagnosed with type 1 diabetes, such as the use of self-monitoring of blood glucose, nutrition counselling, newer insulin analogues and CSII with rtCGM or isCGM, as appropriate. Various features, such as cost, comfort and accuracy, are considerations for product choice.

## Strategies to manage different modalities and durations of exercise

Assuming that glycaemic management has been optimised for non-exercise days, the exercise type (aerobic, anaerobic, mixed) and duration will largely dictate the strategies employed for active days [16]. In general, prolonged predominantly aerobic exercise promotes a drop in blood glucose concentration, while more intensive aerobic and anaerobic events promote a glucose rise [16]. The rise in blood glucose during intensive exercise in the fasted state is reproducible and tends to be associated with a rise in lactate [53]. For endurance events, such as marathons and road cycling, athletes often have elevated glucose levels prior to the event, sometimes because of psycho-physiological stress responses [54] or as a purposeful coping strategy to limit the likelihood of developing hypoglycaemia during the event [55]. Typically, carbohydrate consumption is needed to maintain performance and prevent hypoglycaemia in endurance events lasting ≥60 min [16]. More aerobically fit individuals may have higher hypoglycaemic risk during exercise than those who are less fit [56], potentially due to higher absolute power outputs and greater rates of carbohydrate oxidation. Conversely, having insulin at near basal levels or lower typically causes a rise in glucose during burst events, like pole vaulting, power lifting, sprinting or wrestling [57].

## Optimising performance with nutrition

Several evidence-informed nutritional strategies exist to support athletes in various settings [58]. However, for athletes with type 1 diabetes, it is unclear if special or additional considerations are required to optimise performance. Like athletes without diabetes, those with diabetes follow the full spectrum of carbohydrate intake strategies, depending on their activity and training regimens.

### Carbohydrate intake

While some athletes use carbohydrate counting to determine meal- and snack-based insulin-dose adjustments, this procedure often lacks precision, particularly with high-carbohydrate feeding [59]. Moreover, carbohydrates with differing glycaemic indices and mixed meals make this practice difficult. If exercise occurs soon after a meal, glucose disposal from the meal may be stimulated by both insulin-dependent and insulin-independent signalling [60]. Although high-glycaemic index meals/snacks generally increase insulin resistance in people without diabetes [61], carbohydrate loading pre exercise and/or carbohydrate feeding during competition with simple sugars is feasible [43] and likely to be important for performance and glycaemic management during competition and training.

**Fig. 4:**
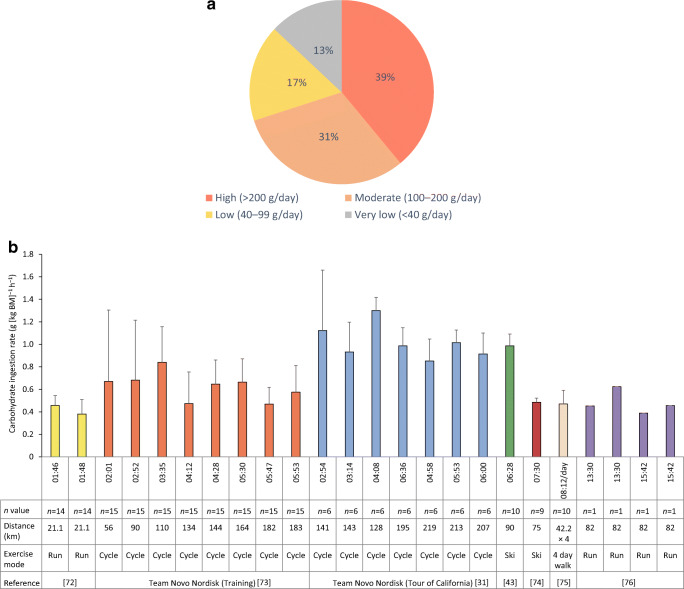
Carbohydrate intake patterns in athletic individuals with type 1 diabetes. (**a**) Daily macronutrient intake from carbohydrates in 252 active adults with type 1 diabetes and of varying athletic level who were surveyed about their carbohydrate intake patterns. Approximately 40% self-reported carbohydrate consumption within the current acceptable macronutrient distribution range of 45–65% of energy intake (>200 g/day) [109], whereas ~30% consciously moderated their carbohydrate intake (100–200 g/day), typically by avoiding starchy or sugary foods. The remainder described following either a low-carbohydrate diet (17% of participants; ~40–99 g/day) or a very-low-carbohydrate diet (13% of participants), with a meal plan of carbohydrate intake of <40 g/day (S. R. Colberg, unpublished data). (**b**) Carbohydrate intake rates during endurance training and competition events: Data are from select studies and were analysed by R. M. Bracken [31, 43, 72–76]. The *x*-axis provides information on exercise duration (h:min) and also profiles the exercise distance and mode used in each of the published studies. Mean carbohydrate intake rate across the studies analysed was 0.70 ± 0.26 g [kg body mass]^−1^ h^−1^ (50 ± 18 g/h). BM, body mass. This figure is available as part of a downloadable slideset

According to self-report, some athletes with type 1 diabetes adopt low or moderate carbohydrate diets to improve glycaemic management (Fig. 4a). It is currently unclear if this dietary approach has an impact on performance. Good long-term glucose management improves performance in athletes with type 1 diabetes: those with lower HbA_1c_ levels (~48 mmol/mol [6.5%]) have superior cardiorespiratory fitness and pulmonary function than those with higher HbA_1c_ levels (~62 mmol/mol [7.8%]) [62]. However, it is unclear if achieving this via restricted carbohydrate feeding, rather than by administering more insulin or by some other means, may compromise endurance performance and/or increase hypoglycaemia or ketoacidosis risk [63].

Muscle glycogen replenishment following exercise requires effective blood glucose management and balancing of carbohydrate intake with insulin dosing. In one study, moderate carbohydrate intake (50% of total energy) was superior to high carbohydrate intake (59% of total energy) for glycogen replenishment, glycaemic management and performance [64]. If carbohydrate intake is limited after prolonged exercise, restoring muscle glycogen levels is likely to take longer [65], which may increase nocturnal hypoglycaemia risk [63]. Co-ingesting protein with moderate amounts of carbohydrate (e.g. 0.8 g kg^−1^ h^−1^) post exercise may provide a feasible option for normal muscle glycogen repletion, while still balancing blood glucose levels [66]. However, high dietary protein intake does not appear to increase muscle glycogen repletion rates further in those already consuming enough carbohydrate [67].

During exercise, carbohydrate requirements depend on the use of insulin or other medications, exercise timing, activity undertaken and starting blood glucose levels. Because of a tendency for lower insulin levels and/or elevation in morning cortisol and growth hormone levels, training before breakfast may require little-to-no carbohydrate ingestion during activity, as compared with afternoon exercise [68]. An elevated pre-exercise blood glucose level in the morning or afternoon reduces carbohydrate intake needs. Participation in resistance exercise [69] and high-intensity interval-type training [53] may not require carbohydrate intake since glucose levels tend not to drop.

Carbohydrate intake and/or insulin reduction is typically required for activities lasting >30 min in a non-fasting state, to prevent hypoglycaemia. For low-to-moderate-intensity aerobic activities lasting 30–60 min that are undertaken when circulating insulin is at basal levels, the intake of small amounts (8–20 g) of carbohydrate may suffice to limit hypoglycaemia, but are not likely to affect performance [70]. With higher circulating insulin exposure due to bolus insulin administration, 30–60 g/h carbohydrate may be needed when the exercise duration lasts >30 min [71]. Carbohydrate intake rates of 0.4 g to 1.3 g carbohydrate per kg body mass per h have been reported for athletes with type 1 diabetes exercising in performance settings lasting ≥60 min (Fig. 4b). These studies found that carbohydrate intake within this range prevented hypoglycaemia and enhanced endurance performance in prolonged exercise [31, 43, 72–76].

### Hydration and electrolyte balance

Adequate hydration during training and competition is required to maintain blood volume and for thermoregulation [77]. Athletes with type 1 diabetes may experience mild to moderate dehydration during exercise if their blood glucose is elevated, which can be exacerbated by the fact that hyperglycaemia increases urinary water loss. Fluid intake during training tends to be higher in type 1 diabetes, as compared with control individuals, perhaps because of elevated thirst caused by hyperglycaemia [78]. In general, plain water or a carbohydrate–electrolyte beverage, depending on glucose level, should be consumed at a rate of ~1 l/h [79].

## Recommendations for rtCGM/isCGM use

rtCGM and isCGM may allow athletes with type 1 diabetes to better manage their glucose levels during training, competition and recovery. When used during prolonged exercise, the initiation of carbohydrate feeding can be based on glucose concentrations (e.g. sensor glucose <8.0 mmol/l), glucose trend arrows and rate of change data [70]. Glucose data should be analysed together with a connected smart pen that can automatically log insulin administration [80], or with pump data [81], to better manage complex situations that may arise due to exercise. With multi-day training, monitoring the AGP can help athletes and clinicians to define achievable blood glucose (and, consequently, performance) goals [82]. Due to the unique challenges of glycaemic management during competition, athletes with type 1 diabetes should engage in several training sessions that closely mimic competition-day conditions to optimise management strategies.

**Fig. 5:**
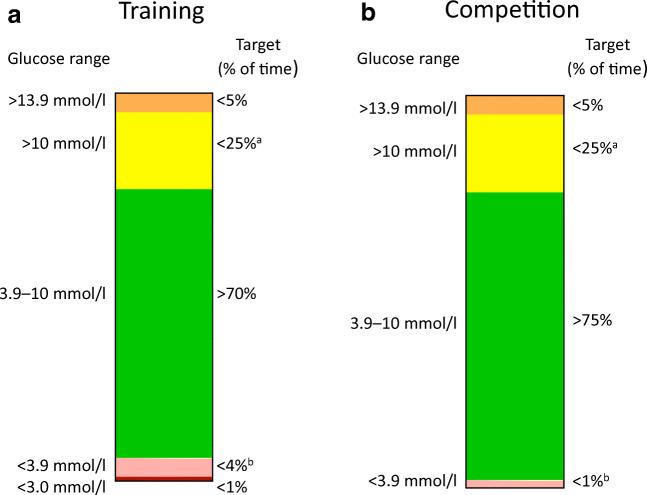
Proposed CGM-based targets for athletes with type 1 diabetes during training (**a**) and competition (**b**). Targets for training days are based on the international consensus [83], while the targets for competition are based on the opinion of the authors. Individual targets should be set by the individual’s healthcare provider with consideration of several variables, including age, duration of diabetes, diabetes-related complications and level of hypoglycaemia awareness. In both training and competition in individuals aged <25 years, if the HbA_1c_ goal is <58 mmol/mol [7.5%], then the TIR target should be set to ~60% but a goal of <4% time below target range (<3.9 mmol/l glucose) should be maintained. ^a^Includes percentage of values >13.9 mmol/l. ^b^Includes percentage of values <3.0 mmol/l. This figure is available as part of a downloadable slideset

The glycaemic targets for health and performance of athletes with type 1 diabetes should be individualised. However, we propose that for any training period, athletes should aim for >70% TIR (3.9–10.0 mmol/l), with <4% below 3.9 mmol/l and <1% below 3.0 mmol/l, identical to the recommendations for the type 1 diabetes adult population [83] (Fig. 5). Since hypoglycaemia during exercise can severely impact performance and, potentially, heart rate variability [84], athletes should aim for <1% time below target and >75% TIR during competition. Reducing glycaemic variability, as measured by a coefficient of variation of ≤36% for CGM values, is also recommended since values above this threshold appear to correlate with increased hypoglycaemia risk [85]. While we acknowledge that these targets are ambitious, they may be achievable with newer technologies and dedication.

## Additional considerations

Many competing athletes deal with additional factors that can affect performance. Poor recognition of hypoglycaemia, travelling, optimisation of body weight and/or menstrual cycle variations in insulin sensitivity are a few factors that may have an impact on glucose control and performance.

### Hypoglycaemia unawareness

Individuals with type 1 diabetes often develop impaired awareness of hypoglycaemia, which increases the risk for a severe hypoglycaemic event by approximately sixfold [86]. Active individuals may be at an elevated risk for developing impaired hypoglycaemia awareness and counterregulatory failure during exercise: routine exercise blunts counterregulation during a hypoglycaemic event [87], which may be a form of habituation. Altering the training exposure to a novel stimulus, such as high-intensity interval training, may help dishabituation and may improve both hypoglycaemia symptom recognition and counterregulation [88].

### Weight management

Sports like gymnastics and cycling require low body weight (and/or fat mass) for performance, while others benefit from maximised body mass (e.g. Olympic deadlifts). Combat athletes aiming to compete in the lightest weight category possible often must lose weight for pre-event weigh-ins [89]. These athletes typically combine chronic and acute strategies to achieve target weights, including energy restriction and dehydration [90]. Such high-risk practices may increase the likelihood of severe dehydration and, possibly, even death [91]. Safe and effective weight management strategies are possible in athletes with type 1 diabetes. Since insulin is an anabolic/anti-catabolic hormone [18], gradual reductions in both energy intake and insulin daily dose are effective for gradually lowering fat mass without compromising muscle mass and safety. It should be noted that acute episodes of hypoglycaemia are associated with food cravings, which can cause disinhibited eating behaviours [92]. Training in settings of low circulating insulin levels should maximise energy provision and training adaptations without requiring excessive snacking, if weight loss is desired [63].

### The female athlete

Female athletes with type 1 diabetes may have unique glycaemic responses to training and competition depending on the stage of the menstrual cycle that they are currently in, and may have a reduced risk for hypoglycaemia as compared with male athletes [93]. Female athletes should be aware that insulin and carbohydrate needs before and after exercise/training may differ throughout their menstrual cycle. In general, higher blood glucose levels are found during the luteal phase, which is often not fully abolished by increasing basal insulin delivery rate [94]. Since the luteal phase is also associated with high oestrogen levels and rising progesterone levels, hyperglycaemia is more prevalent [95] and an increased reliance on lipids as a fuel source during training and recovery may occur [96]. Moreover, the luteal phase is associated with less muscle glycogen mobilisation during endurance exercise, at least in those without diabetes [97], implying that less carbohydrate intake may be required for post-exercise glycogen replenishment.

### Travel

**Fig. 6:**
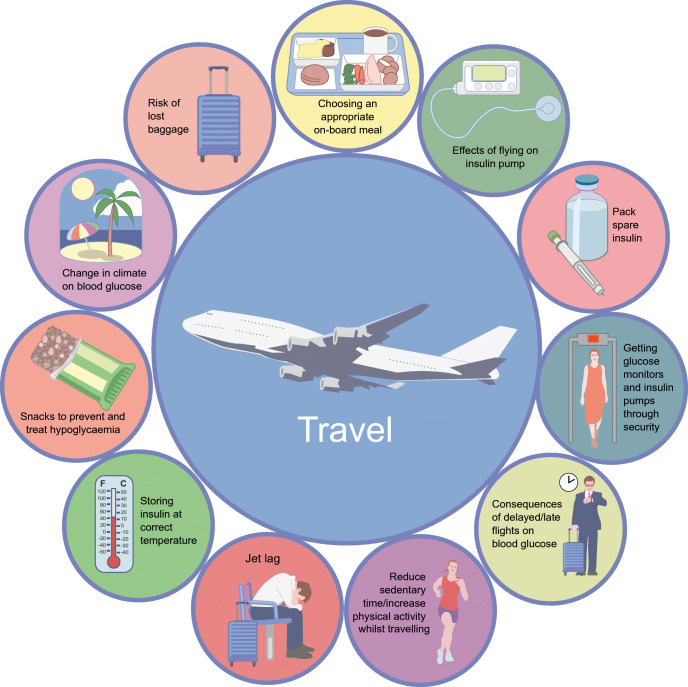
Additional travel considerations for athletes with type 1 diabetes. A summary of practical considerations that an athlete with type 1 diabetes should take into account when travelling for athletic competition. Long-distance travel typically increases sedentary time (not shown), can alter food choices and tends to be associated with risk of hypo- and hyperglycaemia [99]. Increased vigilance around glucose monitoring and insulin-dose alterations, as well as access to healthy dietary options, diabetes supplies and, at least, light physical activity (not shown), should be considered. This figure is available as part of a downloadable slideset

Regular travel, a key part of being a modern-day athlete, can present a significant challenge to athletes with type 1 diabetes. Individuals need to be well prepared for their journey by ensuring they have enough accessible supplies (Fig. 6) [98]. Difficulties may arise from practical decisions about packing insulin properly and bringing spare diabetes-related supplies (e.g. meters, sensors, pumps, needles, glucagon, snacks, etc.) in carry-on luggage. Choosing appropriate travel insurance, dealing with airport security procedures, delayed flights and choosing appropriate on-board meals are also important considerations. When flying long distances and crossing multiple time zones, individuals must develop strategies to adapt to new time zones, limit the effects of jet lag/travel on insulin needs and be hypervigilant to manage blood glucose levels [99]. Athletes should prepare for the possibility of losing diabetes-related supplies, consuming unfamiliar foods, and managing changes in climate and other environmental conditions. If significant time zone changes will occur, those using MDI may need to alter their basal insulin strategy, such as by splitting the basal dose into two doses spaced ~12 h apart before departure [100], or use insulin degludec, which has a long half-life (>25 h) and is more flexible with respect to dose timing than insulin glargine (~12 h half-life) [101].

## Summary

Despite the challenges, athletes with type 1 diabetes continue to excel at all levels of competition, with some even achieving gold medals at the Olympic Games. Several strategies can be implemented to help manage athletes with type 1 diabetes (see Text box). Recent advances in glucose monitoring technologies allow for minute by minute manipulations in insulin administration and nutrient intake to achieve near optimal glycaemic control. In general, athletes with type 1 diabetes perform training and competition with elevated circulating insulin levels and blunted glucagon responses that typically require a high rate of carbohydrate consumption in race events. However, some athletes follow a low-to-moderate-carbohydrate diet on non-race days, which appears to improve overall glycaemic control and preserve muscle glycogen storage capacity. Future research is needed to better define the optimal macronutrient diet for training and competition in these exceptional athletes. Maintaining a high TIR should allow for maximal performance and safety during periods of training, travel and competition.

## Electronic supplementary material

Slideset of figures (PPTX 730 kb)

## Abbreviations

AGP: Ambulatory glucose profile
CGM: Continuous glucose monitoring
CSII: Continuous subcutaneous insulin infusion
isCSM: Intermittently scanned continuous glucose monitoring
MDI: Multiple daily injections
rtCGM: Real-time continuous glucose monitoring
TIR: Time in range
