# Supplementary material for: The competitive athlete with type 1 diabetes
Source: Diabetologia. 2020 Jun 12;63(8):1475–90. doi: 10.1007/s00125-020-05183-8 (PMC7351823; doi:10.1007/s00125-020-05183-8)
Supplement: Supplementary file 1 — (PPTX 730 kb) [file 125_2020_5183_MOESM1_ESM.pptx]

## Slide 1
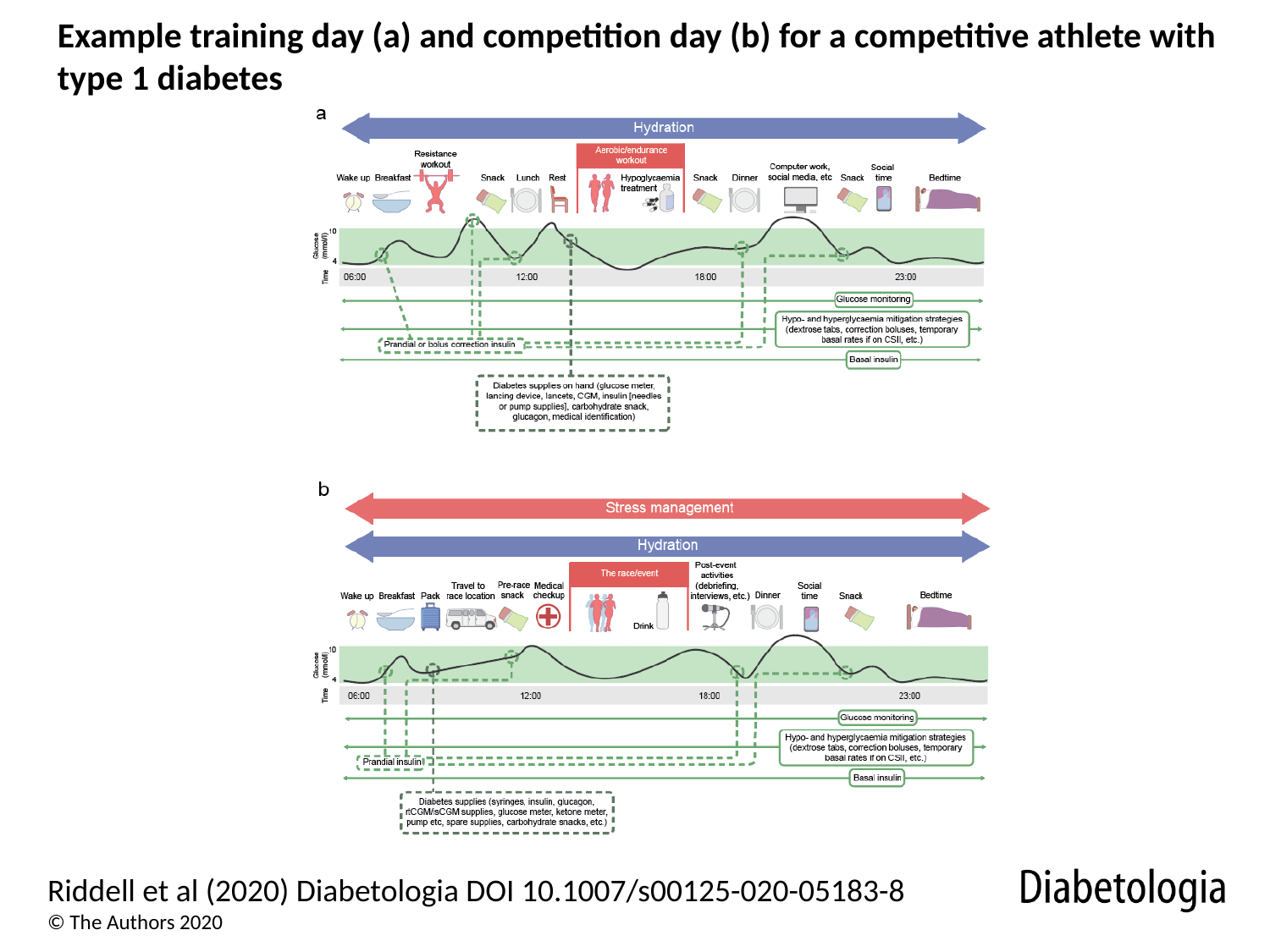

Example training day (a) and competition day (b) for a competitive athlete with type 1 diabetes
Riddell et al (2020) Diabetologia DOI 10.1007/s00125-020-05183-8
© The Authors 2020

## Slide 2
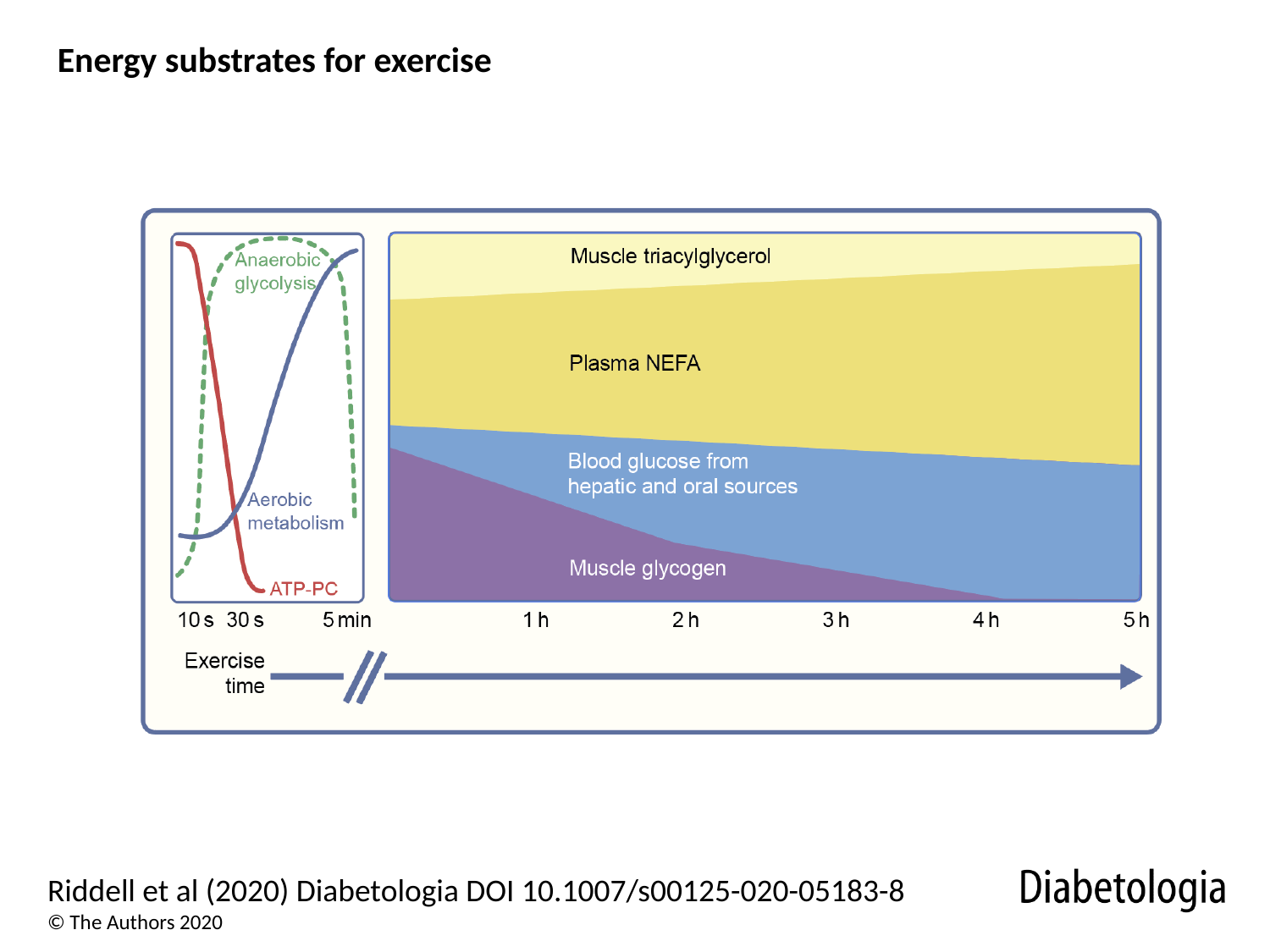

Energy substrates for exercise
Riddell et al (2020) Diabetologia DOI 10.1007/s00125-020-05183-8
© The Authors 2020

## Slide 3
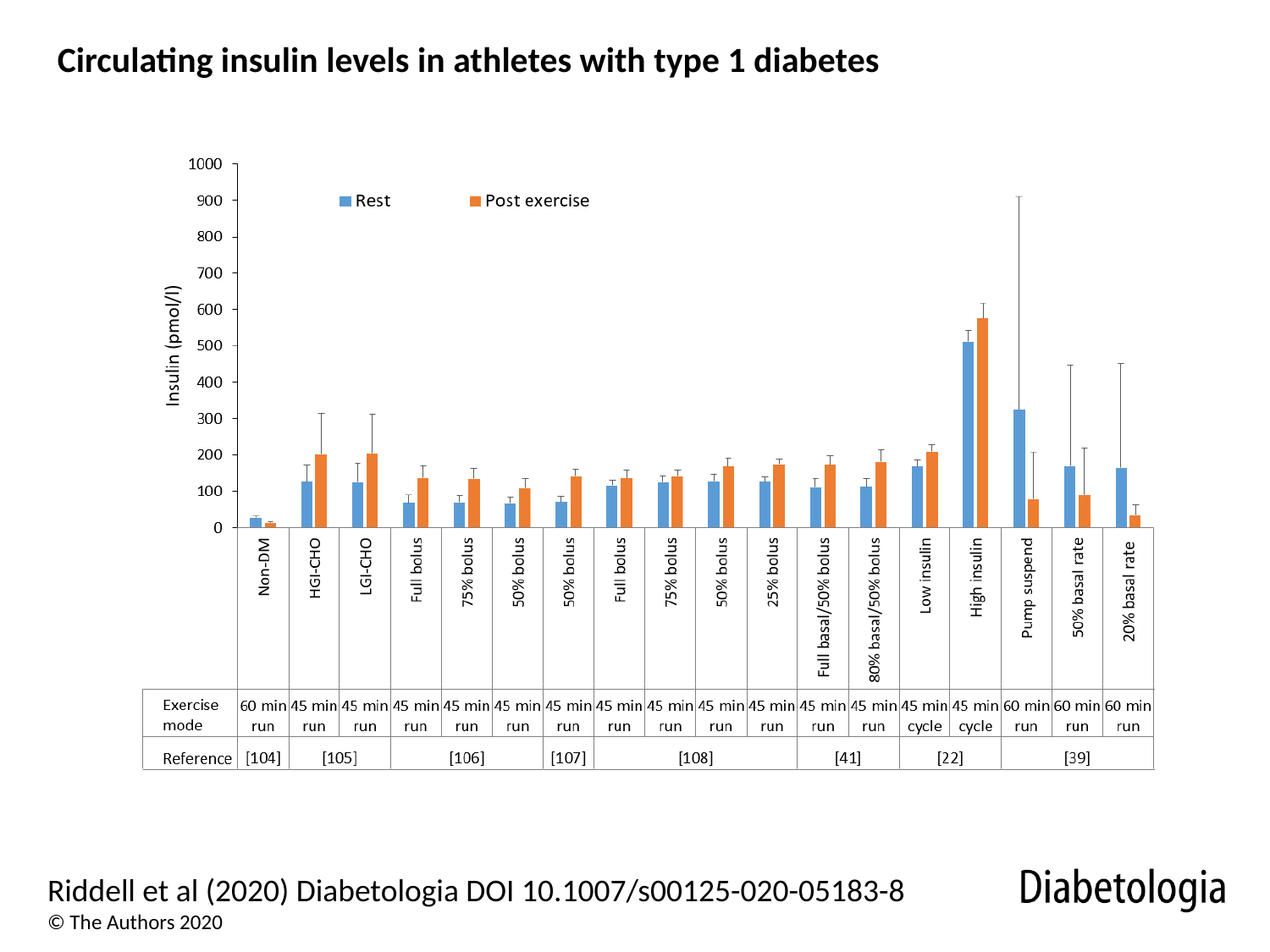

Circulating insulin levels in athletes with type 1 diabetes
Riddell et al (2020) Diabetologia DOI 10.1007/s00125-020-05183-8
© The Authors 2020

## Slide 4
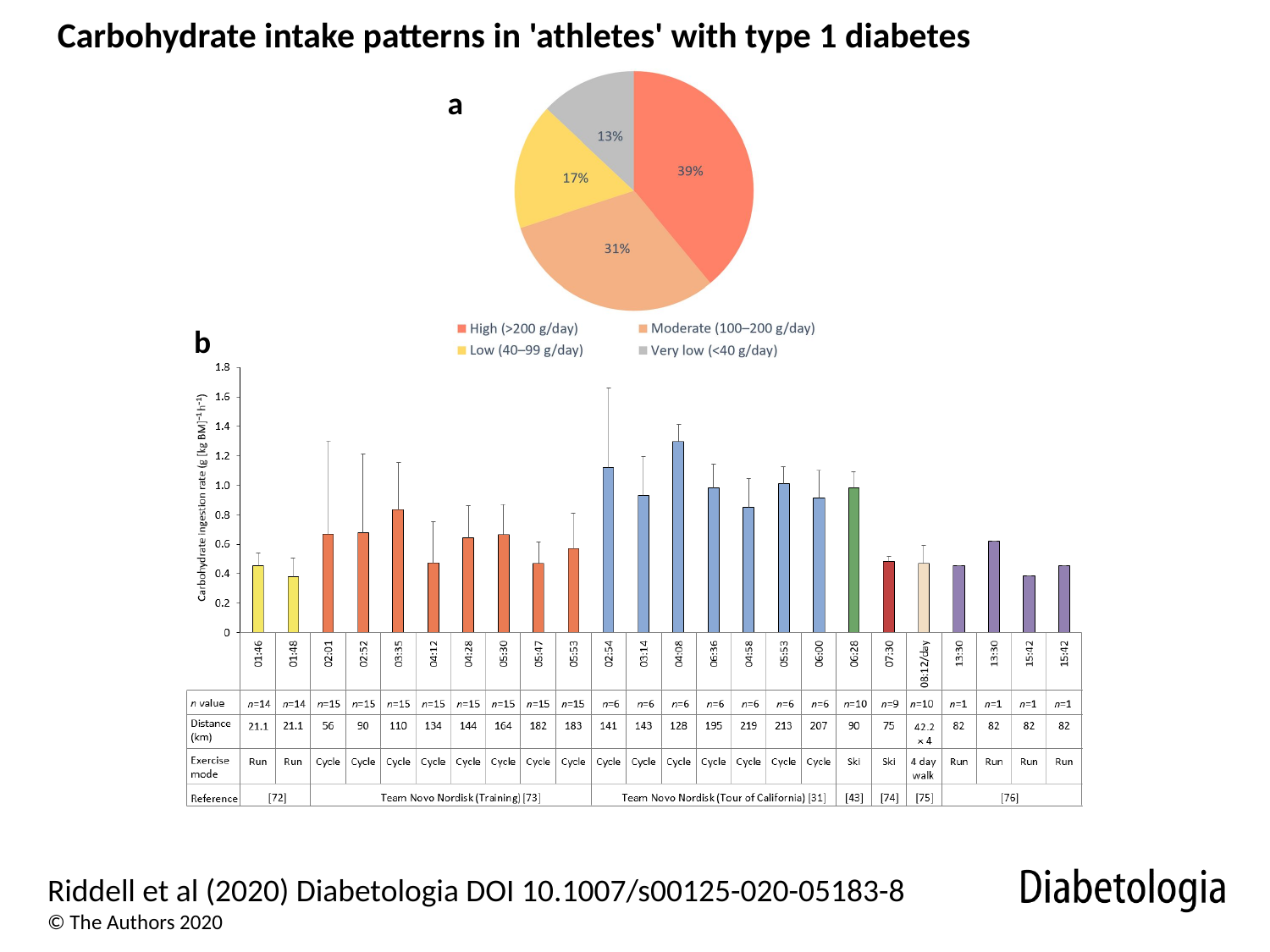

Carbohydrate intake patterns in 'athletes' with type 1 diabetes
a
b
Riddell et al (2020) Diabetologia DOI 10.1007/s00125-020-05183-8
© The Authors 2020

## Slide 5
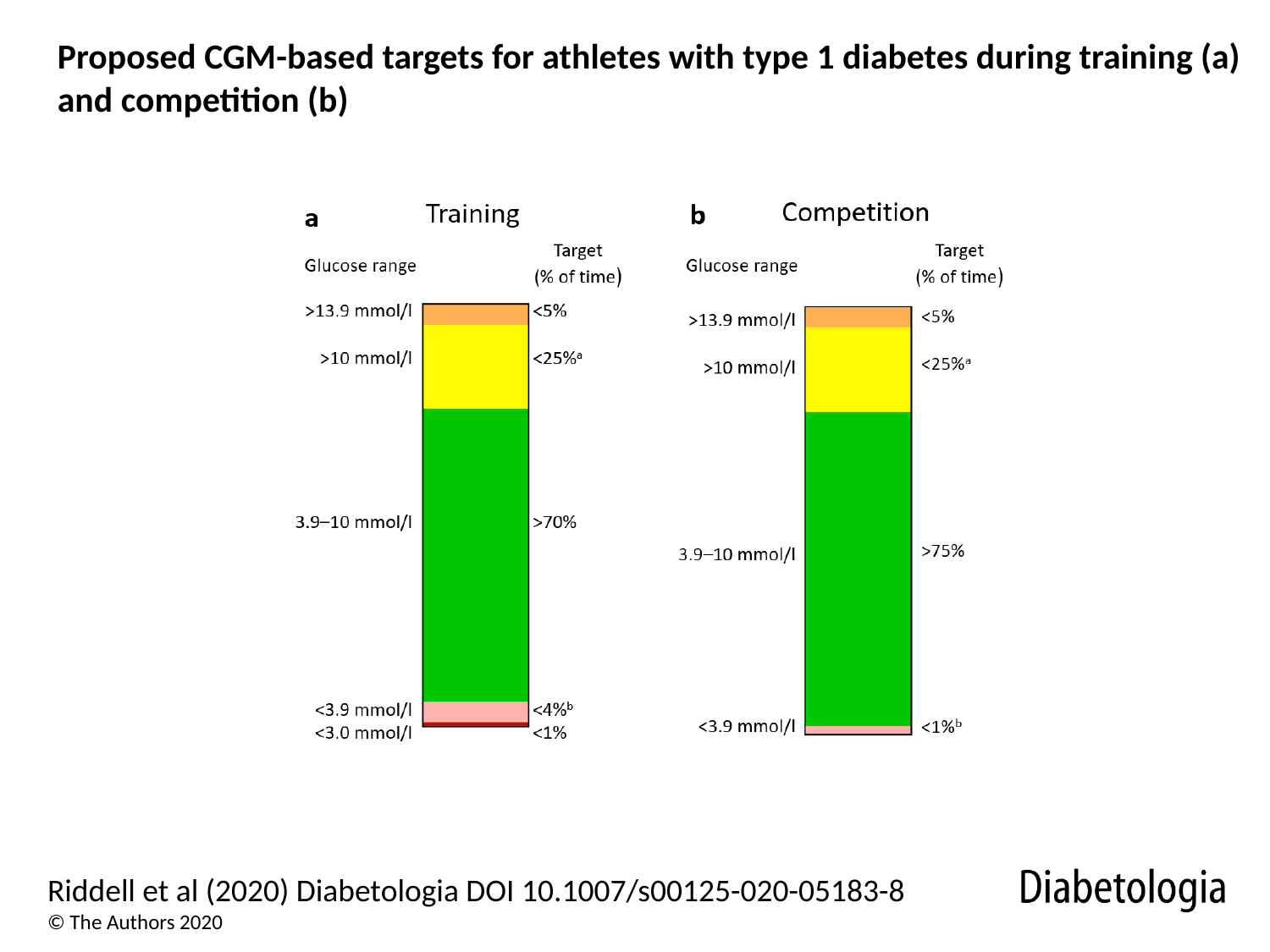

Proposed CGM-based targets for athletes with type 1 diabetes during training (a) and competition (b)
Riddell et al (2020) Diabetologia DOI 10.1007/s00125-020-05183-8
© The Authors 2020

## Slide 6
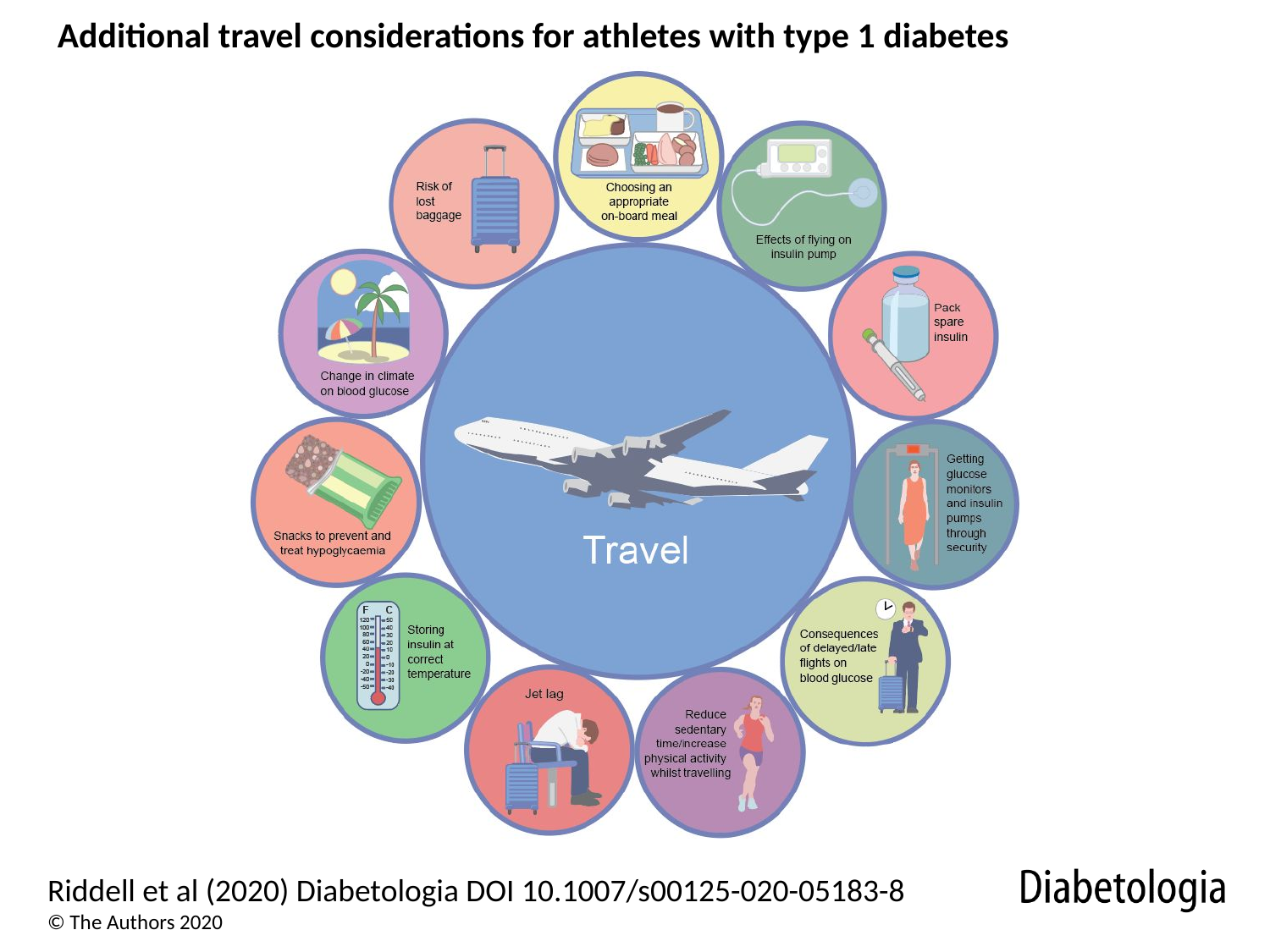

Additional travel considerations for athletes with type 1 diabetes
Riddell et al (2020) Diabetologia DOI 10.1007/s00125-020-05183-8
© The Authors 2020
